# Effective extraction of *Arabidopsis* adherent seed mucilage by ultrasonic treatment

**DOI:** 10.1038/srep40672

**Published:** 2017-01-16

**Authors:** Xianhai Zhao, Lijun Qiao, Ai-Min Wu

**Affiliations:** 1 State Key Laboratory for Conservation and Utilization of Subtropical Agro-bioresources, South China Agricultural University, Guangzhou, China; 2 Guangdong Key Laboratory for Innovative Development and Utilization of Forest Plant Germplasm, South China Agricultural University, Guangzhou, China; 3 College of Forestry and Landscape Architecture, South China Agricultural University, Guangzhou, China; 4 School of Basic Medical Sciences, Shandong University, Jinan, China

## Abstract

The *Arabidopsis* seed coat is composed of two layers of mucilage, a water-soluble non-adherent outer layer and an adherent inner layer. The non-adherent mucilage can easily be extracted by gentle shaking. However, adherent mucilage is extremely difficult to dissociate from the seed coat. Despite various treatments to extract the adherent mucilage, including EDTA, ammonium oxalate, dilute alkali or acid washes, most of it remains on the seed coat. Here, we show for the first time the extraction of almost all of the adherent mucilage from the *Arabidopsis* seed coat. Our results demonstrate that ultrasonic treatment was able to extract the adherent mucilage effectively within 20 seconds. Adherent mucilage, like non-adherent mucilage, is mainly composed of rhamnogalacturonan I (RG I). The crystalline cellulose content in adherent mucilage was measured as 3.7 mg g^−1^ of dry seed. Compared with non-adherent mucilage, the adherent mucilage exhibits relatively stable levels of sugar under various environmental conditions. In all cases, adherent mucilage showed higher levels of sugar than non-adherent mucilage. The cell wall remnant could associate with the adherent mucilage, which could prevent the extraction of the adherent mucilage. Our results show that ultrasonic treatment is an effective method for the quick extraction of *Arabidopsis* adherent mucilage with little effort.

After fertilization in plants, cells of the ovule integuments specialize in to multiple cell layers that form tissues of the seed coat. In some species, including members of the *Brassicaceae, Solanaceae, Linaceae* and *Plantaginaceae*, a large amount of pectinaceous mucilage accumulates during this process^1^. Upon imbibition, the pectinaceous mucilage liberates and envelops the seed to form a gel-like capsule. The physiological role of mucilage remains an enigma. It is thought to help seed dispersal, control germination and withstand biotic and abiotic stress^2^,^3^.

*Arabidopsis* mucilage is composed of two layers, a water-soluble non-adherent outer layer and an adherent inner layer. The non-adherent layer can diffuse in water with gentle shaking and shows no obvious structure. Pectin rhamnogalacturonan I (RG I) is the major component of the non-adherent mucilage and comprises 80–90% of the total non-adherent mucilage^4^,^5^. In contrast, the adherent mucilage is strongly adherent to the seed coat and cannot be removed by agitation, enzymatic digestion or harsh chemical treatments^5^,^6^,^7^. The adherent mucilage is primarily comprised of RG I, but also contains cellulose, galactan, xylan, arabinan and homogalacturonan (HG)^4^,^5^,^8^.

Although the *Arabidopsis* seed coat mucilage is not essential for seed viability or germination, it represents an excellent genetic model for studying carbohydrate secretion, secondary cell wall formation and regulation. Forward and reverse genetic studies have revealed tens of genes involved in mucilage formation and *Arabidopsis* seed coat mucilage has become a very promising model system to explore secondary cell wall polysaccharides synthesis (e.g. cellulose, xylan and HG)^9^. However, adherent mucilage is extremely difficult to remove from the seed coat, which prevents an intact view of the mucilage from being displayed.

The objective of this study was to develop a simple, rapid method to effectively extract adherent mucilage. Adherent mucilage was extracted using various methods to compare efficiency. Ultrasonic treatment demonstrated the best extraction efficiency with the shortest amount of time and labor expended. *In situ* labeling showed that the inner mucilage disappeared almost completely after ultrasonic treatment. Sequential ultrasonic treatments were also performed to investigate optimal extraction times. A detailed study of the mucilage composition and observations by microscopy were undertaken.

## Results

### Ultrasonic treatment to effectively extract the adherent mucilage

**Figure 1:**
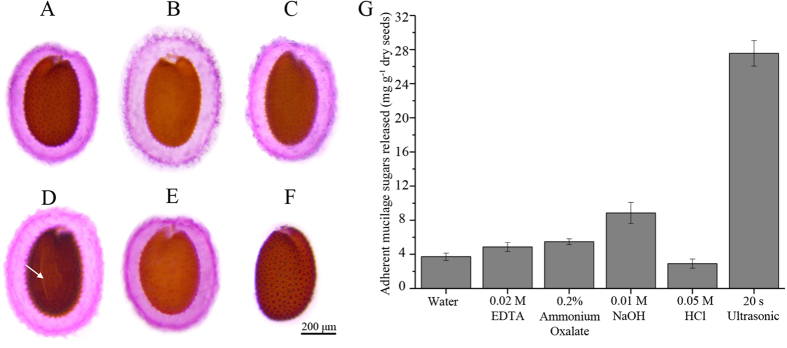
Ruthenium red staining and sugar content of mucilage after different extraction methods. (**A**–**E**) Seeds stained after vigorous agitation in water (**A**), 0.02 M EDTA (**B**), 0.2% (w/v) ammonium oxalate (**C**), 0.01 M NaOH (**D**), 0.05 M HCl (**E**) for 1 hour at 40 °C. (**F**) Seeds stained after ultrasonic treatment in water for 20 seconds at room temperature. The arrow shows the rupture after alkali treatment. Scale bar: 200 μm. (**G**) Adherent mucilage recovered with different extraction methods. Error bars represent SE of three biological replicates.

**Figure 2:**
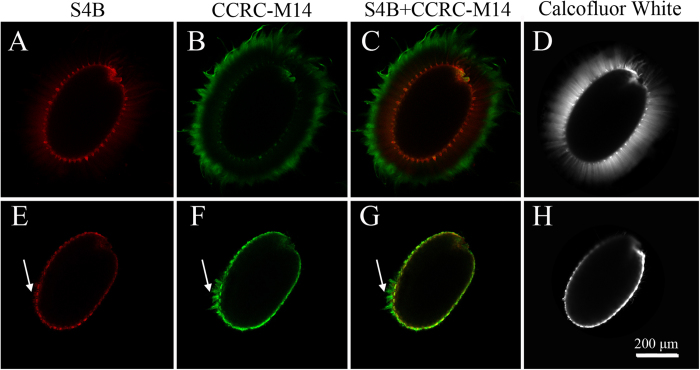
Visualization of mucilage before (**A**–**D**) and after 20 seconds of ultrasonic treatment (**E**–**H**). The arrow shows the cellulose and RG-I residues after 20 seconds of ultrasonic treatment. Scale bar: 200 μm.

As observed previously by Macquet, *et al*.^5^ and as seen in our results (Fig. 1), after vigorous vortexing with either EDTA, ammonium oxalate, dilute alkali or acid, adherent mucilage attached to the seed coat did not show any clear reduction. Alkali treatment resulted in a small portion of the seed coat rupturing, resulting in the leakage of polysaccharides into the extraction buffer (Fig. 1D, arrow). In contrast, ultrasonic treatment for only 20 seconds leads to the disappearance of almost all the mucilage (Fig. 1F). We also observed a reduction in the total volume of the seeds, which was attributed to the removal of the adherent mucilage (Supplemental Figure S1). An examination of the amount of released sugar also showed that chemical agents were only able to extract a small fraction of adherent mucilage, whereas ultrasonic treatment extracted significantly more (up to 6-fold) adherent mucilage (Fig. 1G). To determine whether mucilage remained on the seeds after ultrasonic treatment, immunolabeling and chemical staining was performed. CCRC-M14 and Pontamine Fast Scarlet 4B (S4B) have been shown to bind unbranched RG I and cellulose, respectively. Calcofluor White stains cellulose, callose and other weakly substituted β-glycans. All three approaches showed strong signals with the adherent mucilage layers in the non-ultrasonic treated seeds, which was in accordance with previous research (Fig. 2A–D)^7^,^10^. However, after 20 seconds of ultrasonic treatment, only trace RG I and cellulose signals were observed (Fig. 2E–H), which indicated that the ultrasonic treatment extracted almost all of the adherent mucilage. In addition, 1 minute of the ultrasonic treatment showed no significant influence on seed germination (Supplemental Figure S2). This indicated that even after prolonged ultrasonic extraction, the seeds retained their ability to germinate.

### A 20 second ultrasonic treatment is sufficient for adherent mucilage extraction

**Figure 3:**
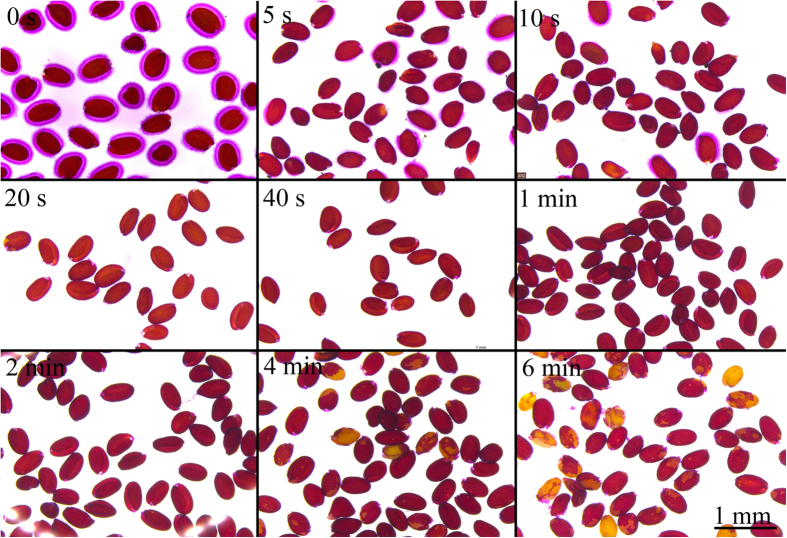
Ruthenium red staining of seed with different ultrasonic treatment time. Scale bar: 1 mm.

**Figure 4:**
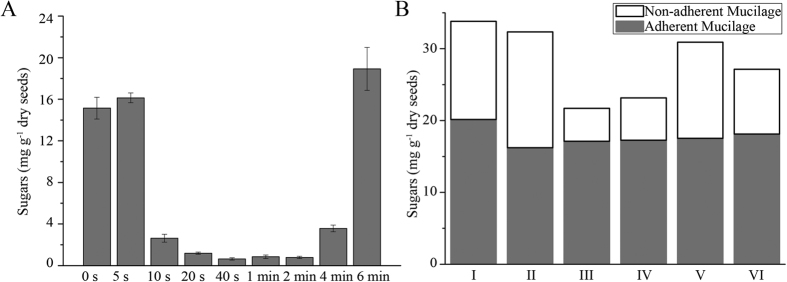
(**A**) Sugars present in total seed that had been extracted following sequential ultrasonic treatment. (**B**) The non-adherent mucilage and adherent mucilage content extracted from six different seeds collection.

To determine the most efficient extraction time using ultrasonic treatment, sequential treatments were performed (Fig. 3). Ruthenium red staining results showed that within 20 seconds of treatment, almost all seeds that were tested lost their mucilage (Fig. 3D), whereas after 4 minutes of treatment, the seed coat began to rupture (Fig. 3H). As observed with ruthenium red staining, after a 20 second treatment, only a small amount of sugar was released in the following ultrasonic treatments (Fig. 4A). However, after 4 minutes of treatment, the sugar release increased. This is likely due to the breaking of the seed coat, causing sugars to be released from the cotyledon and embryo.

A breakdown of the mucilage proportions revealed that non-adherent mucilage comprised 15.56 mg g^−1^ of dry seed whereas the adherent mucilage comprised 21.66 mg g^−1^ of dry seed (Fig. 4B). Since mucilage content was influenced by growing conditions^1^, we analyzed the mucilage content using an additional six independent seed collections planted under different conditions (Fig. 4B). Non-adherent mucilage was 4.60 to 16.10 mg g^−1^ of dry seed. However, the adherent mucilage was 16.23 to 20.14 mg g^−1^ of dry seed. All six lines contained more adherent mucilage, whereas the amount of non-adherent mucilage fluctuated compared to adherent mucilage.

### The mucilage composition

Monosaccharide composition analysis indicated that in both non-adherent and adherent mucilage, GalA and Rha were the major sugars (Table 1). This was consistent with previous results showing that RG I is the major constituent of mucilage^5^,^11^. The adherent mucilage exhibited more sugars derived from hemicellulose, such as Xyl, Gal and Man, which indicates that the adherent mucilage consisted of more hemicellulose than the non-adherent mucilage. GalA and Rha occupied 92.23% molar ratio of the non-adherent total sugar, which was within the previously reported range of 89.71% to 93.99% (Supplemental Table S1)^4^,^5^,^7^,^10^,^12^,^13^. GalA and Rha occupied 82.73% molar ratio of the adherent total sugar, which was also within the previously reported range of 61.50% to 91.49% (Supplemental Table S1).

The crystalline cellulose content in adherent mucilage was measured as 3.7 mg g^−1^ of dry seed, which was higher than the cellulose enzymatic hydrolysis method^5^. However, consistent with previous studies, hardly any crystalline cellulose was detected in non-adherent mucilage. As previously reported, trace protein was detected in the total mucilage (data not shown)^5^.

### FTIR spectroscopy of the mucilage

**Figure 5:**
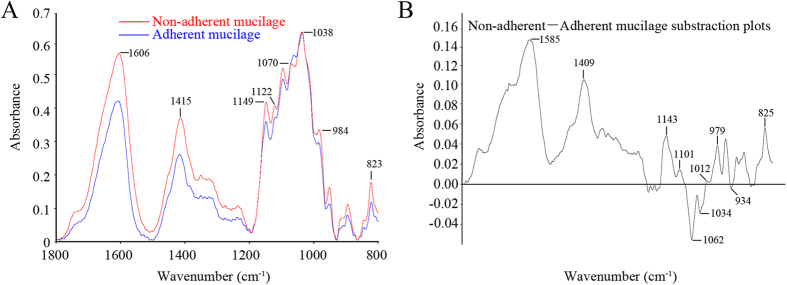
Fourier Transform Infrared Spectroscopy (FTIR) analysis of non-adherent mucilage and adherent mucilage. (**A**) Average spectra of non-adherent mucilage and adherent mucilage. The non-adherent and adherent mucilage have peaks characteristic of RG I (1149, 1122, 1070, 1038, 984, 823 cm^−1^) and peaks at 1606 and 1415 cm^−1^ are carboxylate ion stretches from the galacturonic acid. (**B**) Digital subtraction of an average adherent mucilage spectrum from an average non-adherent mucilage spectrum. The positive peaks at 1143, 1101, 1012, 979, and 825 cm^−1^ indicate that a higher proportion of RG I was present in non-adherent mucilage. The negative peaks at 1062, 1034, and 934 cm^−1^ indicate that a higher proportion of cellulose was present in adherent mucilage. In addition, the positive peaks at 1585 and 1409 cm^−1^ indicated an increased proportion of carboxylate ions in non-adherent mucilage.

Fourier Transform Infrared Spectroscopy (FTIR) is an informative diagnostic tool to detect mucilage structures based on signature peaks^4^,^8^,^14^,^15^. An analysis of the water-soluble non-adherent mucilage and ultrasonic treatment extracted adherent mucilage was performed by FTIR spectroscopy (Fig. 5A). Previous work has indicated that RG I gives rise to peaks in the infrared spectrum at 1150, 1122, 1070, 1043, 985, 823 cm^−1^ 15. Both the non-adherent and adherent mucilage had peak characteristics of RG I (1149, 1122, 1070, 1038, 984, 823 cm^−1^), as well as peaks at 1606 and 1415 cm^−1^, which have been shown to be carboxylate ion stretches derived from galacturonic acid. Consistent with the monosaccharide analysis, this indicates that the mucilage was mainly comprised of components found in RG I. To reveal differences in composition between non-adherent and adherent mucilage, FTIR subtraction spectra representing proportional differences between the two samples were generated (Fig. 5B). The positive peaks at 1143, 1101, 1012, 979, and 825 cm^−1^ indicate that a higher proportion of RG I was present in non-adherent mucilage. The negative peaks at 1062, 1034, and 934 cm^−1^ indicate that a higher proportion of cellulose was present in adherent mucilage. In addition, the positive peaks at 1585 and 1409 cm^−1^ indicated an increased proportion of carboxylate ions in non-adherent mucilage. These results corroborate the above monosaccharide composition analysis, showing that the adherent mucilage contained higher levels of cellulose and hemicellulose.

### Scanning electron microscopy (SEM) and optical microscopy of seeds

**Figure 6:**
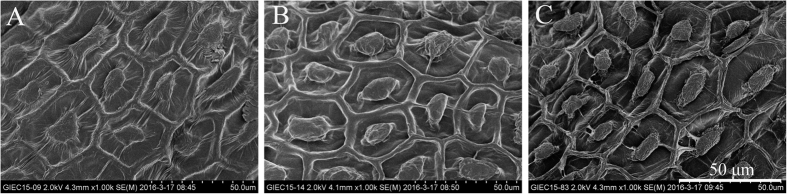
Scanning electron micrograph of intact dry seeds (**A**), air dried seeds after non-adherent mucilage extracted (**B**), air dried seeds after 20 seconds ultrasonic treatment (**C**). Scale bar: 50 μm.

To determine the structure of the seed surface, scanning electron microscopy (SEM) analysis was performed on selected samples (Fig. 6). SEM analysis of intact dry seeds revealed an epidermal layer of hexagonal cells with thickened radial cell walls and a central, volcano-shaped structure known as the columella (Fig. 6A)^1^. After the non-adherent mucilage was extracted and dried, the epidermal primary cell wall was disrupted and the ridge structure disappeared (Fig. 6B). The trough structure was more apparent and the radical cell wall was smoother. This could be attributed to the adherent mucilage, which was spread on the epidermal cell more evenly after imbibition. However, after 20 seconds of ultrasonic treatment, most of the adherent mucilage was extracted and the seed surface appeared rough (Fig. 6C).

Optical microscopy was also used to inspect the seed surface structure. When shaken in ethanol, the hydrophilic mucilage could not absorb ethanol and only the columella was visible (Supplemental Figure S3A,D). When shaken in water, the outer cell walls ruptured and cell wall residues were attached to the columella (Supplemental Figure S3B,E). Even after vigorous shaking overnight, the cell wall remnants remained attached to the columella like the adherent mucilage. However, after 20 seconds of ultrasonic treatment, which resulted in the removal of the adherent mucilage, no cell wall residues remained attached to the columella (Supplemental Figure S3C,F). After each ultrasonic treatment, the cell wall residues were always removed along with the adherent mucilage.

## Discussion

*Arabidopsis* seed coat mucilage represents a very promising model system to explore secondary cell wall polysaccharides biosynthesis. Once extruded, the non-adherent outer layer mucilage is easily removed by gently shaking in water. However, the adherent mucilage is recalcitrant to chemical agents and vigorous shaking. In this study, we showed that short pulses of ultrasonic treatment could effectively extract the adherent mucilage. After extraction of the non-adherent mucilage, the ultrasonic method was able to extract the highest amount of adherent mucilage when compared to treatment by chemical agents. Only trace mucilage could be detected by chemical staining and immunolabeling assays of seed coats after 20 seconds of ultrasonic treatment, which indicated that almost all of the adherent mucilage had been extracted. By using sequential ultrasonic treatment, we showed that 20 seconds is sufficient for the extraction procedure.

In this study, the ultrasonic method was able to extract a higher proportion of adherent mucilage. Seeds collected from different planting conditions showed varying levels of adherent mucilage, which exhibited low levels of fluctuation. In contrast, the non-adherent mucilage showed a high level of fluctuation, which indicates that this part of the mucilage was readily affected by environmental conditions and its composition is not as important to the seeds compared to the adherent mucilage. This is consistent with the fact that this part of the mucilage is easily removed.

As seen in Supplemental Table S1, the adherent mucilage sugar composition fluctuated significantly compared with the non-adherent sugar composition. This could be attributed to the characteristics of the extraction agents. Using water as the extraction buffer, GalA and Rha represent over 91.49% molar ratio of the adherent sugar^16^. This means that water could be used to extract the RG I component effectively. However, other components such as xylan and cellulose remain in the seed coat after extraction. Using either acid or alkali as the extraction agent, more hemicellulose sugars like galactan, xylan, arabinan and mannan could be extracted. The alkaline treatment resulted in hemicellulose sugar molar ratio ranges from 10.2% to 28.9% (Supplemental Table S1). This means that alkali extraction is effective in determining the hemicellulose composition.

Upon imbibition, the pectinaceous mucilage liberates and envelops the seed by forming a gel-like capsule around the seed. This event is correlated with breakage of the outer tangential cell wall of the epidermal cell^1^. The hydrophilic mucilage expands rapidly upon hydration and leads to the breakage of the cell walls. The adherent mucilage is compact in the seed coat and resistant to extraction. However, after ultrasonic treatment, the remnant cell wall is completely removed with the adherent mucilage (Supplemental Figure S3C,F). This phenomenon may provide an explanation as to why the adherent mucilage is strongly attached to the seed coat; cell wall remnants may associate with the adherent mucilage and prevent it from being released.

A major advantage in using water as the ultrasonic extraction buffer is that the dialysis procedure necessary with the alkali or acid treatment is omitted, which saves significant time and labor. However, after ultrasonic treatment, it appears that much of the adherent mucilage is stripped from the seed coat intact. Thus, after the ultrasonic extraction, the extraction buffer becomes turbid and cannot be readily filtered (0.45 μm), even after vigorous shaking. This phenomenon indicates that the adherent mucilage polysaccharides are cross-linked with each other and form a tight bond. Therefore, if gel permeation chromatography analysis has to be performed, the extraction needs to be treated with alkali or acid to break the linkages of the macromolecular structures.

In conclusion, we show that ultrasonic treatment is an effective method for the extraction of *Arabidopsis* adherent mucilage. This method is able to accomplish the extraction in several minutes and produces the best yield of adherent mucilage thus far reported. The adherent mucilage is extracted in an intact form and likely represents the complete extraction of adherent mucilage from seeds.

## Materials and Methods

### Plant material and growth conditions

The seeds used in this study were from *Arabidopsis thaliana* accession Col-0. Seeds were harvested from soil-grown plants under long-day conditions (16 hours light, 8 hours dark) at 22 °C in Percival I-36VL incubator. For comparing planting conditions on the mucilage content, seeds were collected from different planting conditions.

### Mucilage Extraction

Approximately 5 mg of seed was precisely weighed in 1.5 mL Eppendorf tubes. After hydration for 5 min in 1 mL water with gentle mixing, supernatants were recovered as the non-adherent mucilage. Seeds were rinsed twice with water and 1 mL water was added. A Sonics VCX130PB equipped with a 3 mm probe (60% amplitude) was used for the ultrasonic treatment. The seeds were allowed to settle at the bottom of each tube, and the supernatants were recovered as the adherent mucilage. For sequential ultrasonic treatment, seeds were rinsed twice with water after every treatment. Adherent mucilage was also extracted using water, 0.02 M EDTA, 0.2% (w/v) ammonium oxalate, 0.01 M NaOH, and 0.05 M HCl for 1 hour with vigorous shaking at 40 °C.

### Chemical analysis

Total sugars were determined with a phenol-sulfuric assay based on the method of DuBois, *et al*.^17^. In brief, 300 μL sample was incubated with 150 μL freshly made 5% (v/v) aqueous phenol and 1.5 mL concentrated sulfuric acid for 1 hour at room temperature. A linear response curve was obtained with glucose standards of 0, 10, 20, 40, 60, 80, 100, 150, 200 μg mL^−1^ final concentration. Absorbance was detected at 490 nm and each sample was repeated four times. For the determination of crystalline cellulose content, mucilage extracted from 5 mg seeds was hydrolyzed in 2 M TFA at 121 °C for 1 hour. After centrifugation, the pellets were suspended in 140 μL 70% sulfuric acid for 1 hour. The amounts of crystalline cellulose were quantified with a modified anthrone assay^18^. After the hydrolysis, 420 μL water was added to obtain a 560 μL sample. Glucose standards (0, 10, 20, 40, 60, 80, 100, 150, 200 μg mL^−1^ final concentration) were also prepared with 1/4 diluted sulfuric acid. A total of 1.4 mL ice cold 0.2% anthrone reagent (w/v, freshly prepared) was added to the samples and glucose standards, and then vortex-mixed gently but thoroughly. After incubation in a boiling water bath for 15 min, absorbance was detected at 620 nm and every sample was repeated four times. Protein concentrations were determined by the Bradford method^19^ using a protein assay kit (Thermo Scientific).

### Monosaccharide composition

The non-adherent mucilage and adherent mucilage monosaccharide compositions were analyzed as previously described^20^. In brief, mucilage extracted from 5 mg of seed were hydrolyzed using 2 N trifluoroacetic acid for 2 hours. After evaporating the trifluoroacetic acid, the hydrolysates were derivatized with 1-phenyl-3-methyl-5-pyrazolone and NaOH at 70 °C for 2 hours. HCl was then added for neutralization. The mixture was extracted with dichloromethane 3 times and then analyzed on a ZORBAX Eclipse XDB-C_18_ column (2.1 × 250 mm; Agilent) connected to a Agilent 1200 HPLC System at a constant flow rate of 0.5 mL/min. 5 μL sample was injected, eluted with 70% (v/v) ammonium formate buffer (0.1 M, pH 5.5) and 30% (v/v) acetonitrile and monitored by UV A_245_.

### Visualization of mucilage

After hydration for 5 min in water with gentle mixing, the water phase was removed and the seeds were used for the chemical stain and immunolabeling experiments. For ruthenium red staining, seeds were imbibed with 0.01% (w/v) ruthenium red (Sigma Aldrich) for 5 min. Then, the seeds were washed two times with water and imaged with a Leica M165C stereomicroscope. For Calcofluor White stain, seeds were imbibed with 25 μg mL^−1^ fluorescent brightener 28 (Sigma) for 30 min. Then, the seeds were washed five times with water and imaged with a Zeiss LSM710 confocal microscope (Zeiss) using 405 nm. For RG-I (CCRC-M14) and cellulose (S4B) labeling, seeds were rinsed twice with phosphate-buffered saline (PBS) and blocked with 5% (w/v) skim milk powder in PBS for 1 hour. Seeds were then sequentially incubated with RG I-specific primary antibody CCRC-M14 (1/20 dilution; Complex Carbohydrate Research Center) and fluorescein isothiocyanate-conjugated anti-mouse secondary antibodies (1/50 dilution) in PBS for 1 hour. After wash in PBS and counterstaining for 5 min with 0.01% (w/v) S4B (Direct Red 23, Sigma Aldrich) in 50 mM NaCl, the immunofluorescence was observed using a Zeiss LSM710 confocal microscope (Zeiss) using 490 nm and 552 nm lasers.

### Scanning electron microscopy

Seeds after mucilage extraction were air dried, and dry seeds were mounted on aluminum stubs (Hitachi Ion Sputter E-1010, HITACHI), sputter-coated with 40 nm of gold-palladium and viewed using a Hitachi S-4800 FESEM.

### Fourier transform infrared spectroscopy (FT-IR)

The mucilage samples were freeze-dried. Then, KBr disc standard technique was performed according to methods described in a previous study^21^.

## Additional Information

**How to cite this article:** Zhao, X. *et al*. Effective extraction of *Arabidopsis* adherent seed mucilage by ultrasonic treatment. *Sci. Rep.* **7**, 40672; doi: 10.1038/srep40672 (2017).

**Publisher's note:** Springer Nature remains neutral with regard to jurisdictional claims in published maps and institutional affiliations.

## Supplementary Material

Supplementary Dataset 1

Supplementary Information

## Figures and Tables

**Table 1:** Monosaccharide composition of sequentially extracted mucilage.

	Non-Adherent Mucilage	Adherent Mucilage
Man	0.23 ± 0.06	1.27 ± 0.21
GalA	47.07 ± 1.34	42.69 ± 1.08
Rha	45.16 ± 2.36	40.04 ± 1.66
Non-cellulosic Glc	0.4 ± 0.18	2.51 ± 0.21
Gal	1.17 ± 0.17	6.06 ± 0.05
Ara	1.39 ± 0.08	1.28 ± 0.81
Xyl	4.57 ± 0.69	6.15 ± 0.22
GalA/Rha	1.05 ± 0.08	1.07 ± 0.07

The values are the relative monosaccharide compositions (mol %) of triplicate assays ± SE.
