# Supplementary material for: Effective extraction of Arabidopsis adherent seed mucilage by ultrasonic treatment
Source: Sci Rep. 2017 Jan 16;7:40672. doi: 10.1038/srep40672 (PMC5238429; doi:10.1038/srep40672)
Supplement: Supplementary Information [file srep40672-s2.doc]

***Supplementary Information***

**Effective extraction of *Arabidopsis* adherent seed mucilage by ultrasonic treatment**

Xianhai Zhao^1,2,3^, Lijun Qiao^4^ & Ai-Min Wu^1,2,3*^

^1^State Key Laboratory for Conservation and Utilization of Subtropical Agro-bioresources, South China Agricultural University, Guangzhou, China. ^2^Guangdong Key Laboratory for Innovative Development and Utilization of Forest Plant Germplasm, South China Agricultural University, Guangzhou, China. ^3^College of Forestry and Landscape Architecture, South China Agricultural University, Guangzhou, China. ^4^School of Basic Medical Sciences, Shandong University, Jinan, China.

*Correspondence and requests for materials should be addressed to A.M.W (email: wuaimin@scau.edu.cn)


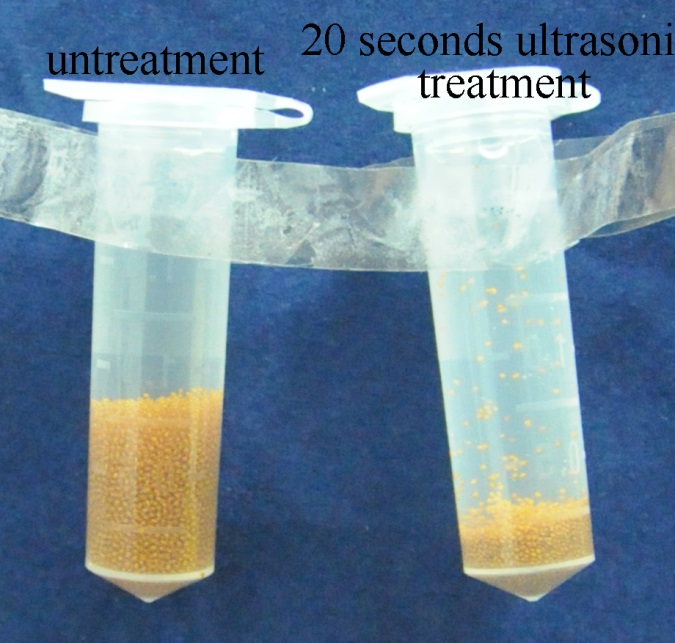


**Supplemental Figure S1.** The volume of 100 mg seeds in 1.5 mL water was reduced significantly after 20 seconds of ultrasonic treatment.


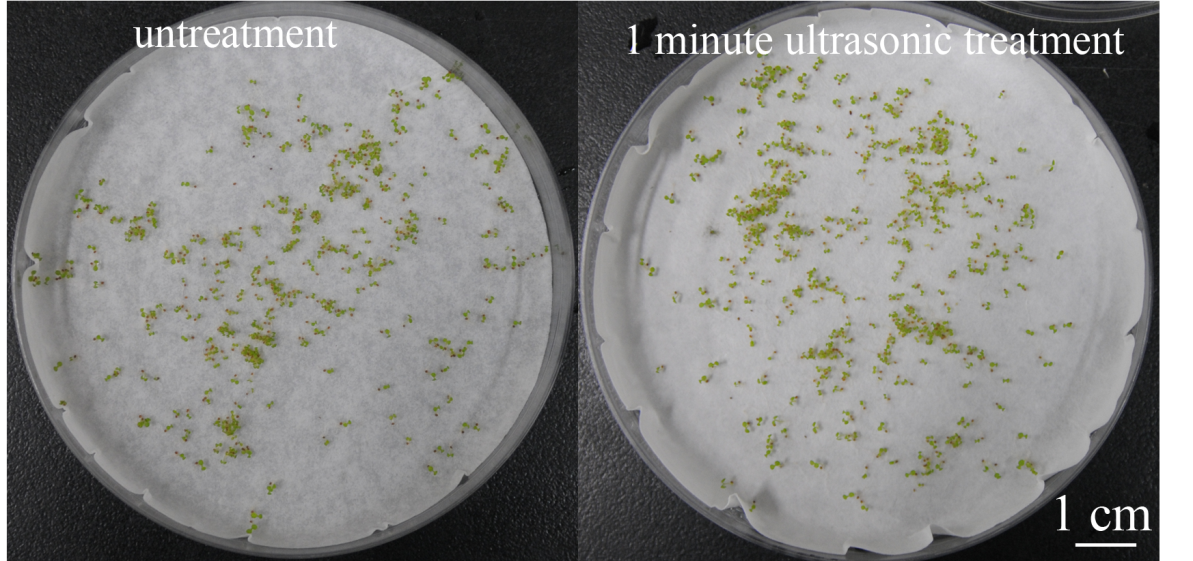


**Supplemental Figure S2.** The seeds with and without ultrasonic treatment were germinated on filter paper. Scale bar: 1 cm.


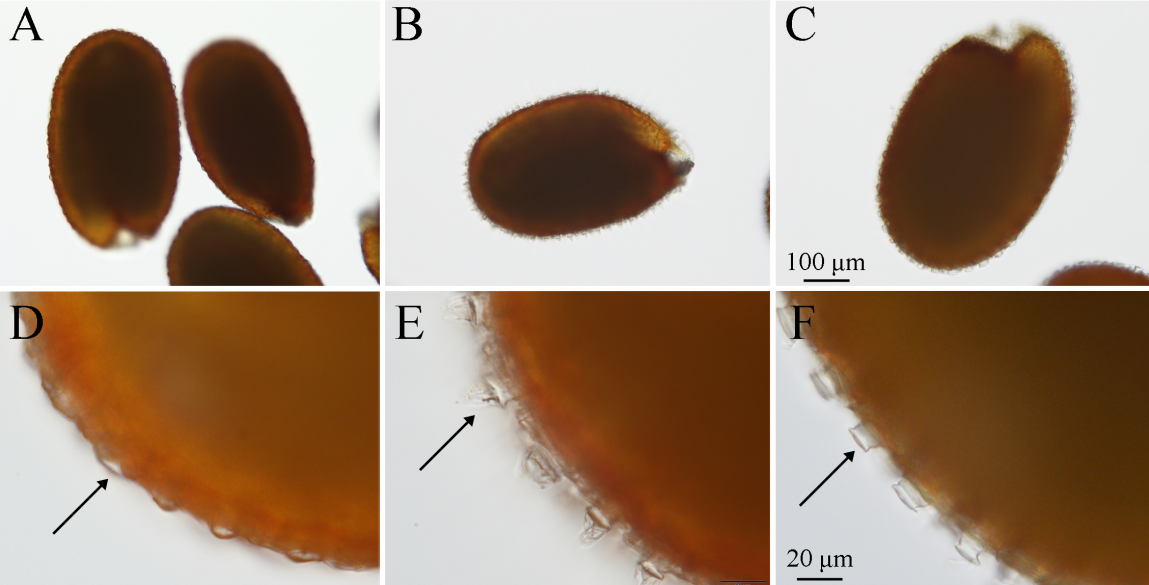


**Supplemental Figure S3.** The outer cell wall of the seed coat. (A, D) Dry seeds were shaken in ethanol for 1 hour. The arrow showed the columellae. (B, E) Dry seeds were shaken in water for 1 hour. The arrow showed the columellae and the cell wall remnant. (C, F) Dry seeds were shaken in water for 5 minutes and ultrasonic 20 seconds. The arrow showed the columellae without the cell wall remnant. Scale bar in A-C : 100 μm, Scale bar in D-F: 20 μm.
